# The Image–Histology Correlation of Subcutaneous mPEG-poly(Ala) Hydrogel-Embedded MIN6 Cell Grafts in Nude Mice

**DOI:** 10.3390/polym15122584

**Published:** 2023-06-06

**Authors:** Jyuhn-Huarng Juang, Chen-Ling Chen, Chen-Wei Kao, Chen-Yi Chen, Chia-Rui Shen, Jiun-Jie Wang, Zei-Tsan Tsai, I-Ming Chu

**Affiliations:** 1 Division of Endocrinology and Metabolism, Department of Internal Medicine and Center for Tissue Engineering, Chang Gung Memorial Hospital, Taoyuan 33305, Taiwan; jenny74513@gmail.com (C.-L.C.); lian8807111@gmail.com (C.-W.K.); je3474@gmail.com (C.-Y.C.); 2 Department of Medicine, College of Medicine, Chang Gung University, Taoyuan 33305, Taiwan; 3 Department of Medical Biotechnology and Laboratory Science, College of Medicine, Chang Gung University, Taoyuan 33305, Taiwan; crshen@mail.cgu.edu.tw; 4 Department of Ophthalmology, Chang Gung Memorial Hospital, Taoyuan 33305, Taiwan; 5 Department of Medical Imaging and Radiological Sciences, College of Medicine, Chang Gung University, Taoyuan 33305, Taiwan; jiunjie.wang@gmail.com; 6 Department of Diagnostic Radiology, Chang Gung Memorial Hospital, Keelung 20401, Taiwan; 7 Molecular Imaging Center, Chang Gung Memorial Hospital, Taoyuan 33305, Taiwan; zeitsan@ms9.hinet.net; 8 Department of Chemical Engineering, National Tsing Hua University, Hsinchu 300044, Taiwan; chuiming123@gmail.com

**Keywords:** mPEG-poly(Ala) hydrogel, MIN6 cells, subcutaneous transplantation, graft imaging, graft histology

## Abstract

Previously, we have successfully used noninvasive magnetic resonance (MR) and bioluminescence imaging to detect and monitor mPEG-poly(Ala) hydrogel-embedded MIN6 cells at the subcutaneous space for up to 64 days. In this study, we further explored the histological evolution of MIN6 cell grafts and correlated it with image findings. MIN6 cells were incubated overnight with chitosan-coated superparamagnetic iron oxide (CSPIO) and then 5 × 10^6^ cells in the 100 μL hydrogel solution were injected subcutaneously into each nude mouse. Grafts were removed and examined the vascularization, cell growth and proliferation with anti-CD31, SMA, insulin and ki67 antibodies, respectively, at 8, 14, 21, 29 and 36 days after transplantation. All grafts were well-vascularized with prominent CD31 and SMA staining at all time points. Interestingly, insulin-positive cells and iron-positive cells were scattered in the graft at 8 and 14 days; while clusters of insulin-positive cells without iron-positive cells appeared in the grafts at 21 days and persisted thereafter, indicating neogrowth of MIN6 cells. Moreover, proliferating MIN6 cells with strong ki67 staining was observed in 21-, 29- and 36-day grafts. Our results indicate that the originally transplanted MIN6 cells proliferated from 21 days that presented distinctive bioluminescence and MR images.

## 1. Introduction

Individuals with type 1 diabetes are insulin deficient, caused by the destruction of pancreatic β-cells [1]. Eventually, they are dependent on exogenous insulin which may cause complexicity of hyperglycemia and hypoglycemia. In contrast, a successful whole pancreas or islet transplantation can restore normal blood glucose [2]. Although most human islet transplantation attains insulin independence at one year [3,4], the successful rate gradually decreases with time [5,6]. By now, the islets are infused into the portal vein and engraft in the liver [3,4,5,6]. However, at this site, ischemia and the inflammatory response may impair islets and lead to their early loss [7,8,9]. Other disadvantages include procedure-related complications [3,4,5,6,10,11] and difficulty in the monitoring and retrieval of transplanted islets. Many sites other than the liver have been investigated, including the subcutaneous tissue, muscle, pancreas, spleen, kidney subcapsule, peritoneal cavity, omentum, thymus, testis, cerebral ventricles, bone marrow, gastric submucosa and anterior chamber of the eye [8,9,12]. Particularly, subcutaneous islet transplantation is considered because it is less invasive and the graft is easily monitored and removed [8,9,12]. However, its results have been disappointing, possibly due to poor vascularization and oxygenation at the subcutaneous site [8,9,12,13,14]. 

Biomaterials have been applied in tissue engineering and regenerative medicine [15]. In islet transplantation, biocompatible scaffolds with a collagen–chitosan hydrogel have been shown to improve islet survival at the subcutaneous site by promoting neovascularization [16]. Injectable hydrogels have the advantage of delivering cells with a minimally invasive procedure [15]. Especially, thermosensitive hydrogels receive great attention because they have unique sol-to-gel transition properties that are important in the application of cell and drug delivery [17]. Moreover, polypeptide thermosensitive hydrogels have characteristics of low gelation concentration, varying secondary structure, and reversibility that can meet the specific requirements [18,19,20]. Particularly, ionic polypeptide copolymers have the potential for extended-release drugs and proteins with opposite charges [21,22]. Accordingly, we developed poly(ethylene glycol) methyl ether [(mPEG)-poly(Ala)] hydrogels which were shown to be biocompatible, biodegradable and non-toxic to cells [23].

The MIN6 cells, derived from an insulinoma cell line, have similar characteristics of isolated islets [24] and have been used for the research of β-cells in vitro, in vivo and transplantation [23,25,26,27,28]. Previously, Sobel et al. showed subcutaneous transplantation of MIN6 cells embedded in Matrigel or HyStem-C hydrogels could reduce blood glucose in nude mice [26]. We also demonstrated that the Matrigel and (mPEG)-poly(Ala) hydrogels were feasible for the delivery of MIN6 cells in the subcutaneous space [23,27,28]. To further understand the fate of transplanted cells, we have successfully used noninvasive bioluminescence and magnetic resonance (MR) imaging to monitor grafts of luciferase-transfected and iron oxide-labeled MIN6 cells for up to 64 days after subcutaneous transplantation. Specifically, the bioluminescence images showed a stronger signal in the graft at 22 and 36 days compared to 13 days and the MR signal intensity of the graft turned from hypointensity at day 21 to hyperintensity at day 29. At the end of study, we found tumor growth of MIN6 cells in the graft [28]. To explore the histological evolution of MIN6 cell grafts and correlate it with the above-mentioned image findings, in this study, we examined the graft vascularization, cell growth and proliferation with anti-CD31, SMA, insulin and ki67 antibodies, respectively, at 8, 14, 21, 29 and 36 days after transplantation. Our results reveal a strong correlation between the graft image and histology.

## 2. Materials and Methods

### 2.1. Materials

ʟ-alanine and mPEG (Mn 2000) were purchased from Sigma-Aldrich (St Louis, MO, USA). Trifluoroacetic acid (TFA) and trifluoroacetic acid-d (TFA-d) were purchased from Alfa Aesar (Wall Hill, MA, USA). Ammonium hydroxide solution (28–30%), toluene, hexane, diethyl ether, and 95% ethanol were purchased from Echo Chemicals (Toufen, Miaoli, Taiwan). Dimethyl sulfoxide (DMSO) was purchased from J.T. Baker (Center Valley, PA, USA). Dulbecco’s modified Eagle’s medium (DMEM), fetal bovine serum (FBS), and antimycotic antibiotics were purchased from Gibco (Grand Island, NY, USA). Tetrahydrofuran (THF) was purchased from TEDIA (Fairfield, OH, USA); dichloromethane (DCM), N,N-dimethylformamide (DMF), and chloroform were obtained from AVANTOR (Center Valley, PA, USA) and dried over CaH2 before being used. Chitosan-coated superparamagnetic iron oxide (CSPIO) was prepared with a chemical co-precipitation method [29]. Prussian blue and anti-CD31 antibody were purchased from Thermo Fisher (Cheshire, UK). Anti-Ki67 antibody and anti-alpha smooth muscle actin (SMA) antibody were purchased from Abcam (Cambridge, UK).

### 2.2. Synthesis of L-Alanine N-Carboxyanhydride (NCA-Ala) and mPEG-poly(Ala) Diblock Copolymers

The mPEG-poly(Ala) diblock copolymer was prepared via the ROP of L-alanine-N-carboxyanhydride (NCA-Ala), with mPEG-NH_2_ serving as the macroinitiator [24]. mPEG-NH_2_ was dissolved in 15 mL of toluene, and azeotropic distillation was used to obtain a final volume of 5 mL. A 200 mL solvent containing a mixture of anhydrous chloroform and N,N-dimethylamide (2:1) and NCA-Ala powder were added together. The reaction was maintained at 40 °C for 1 day. The product was then precipitated in ice-cold diethyl ether, solubilized in DMSO, and then dialyzed (Molecular weight cut-off 1000 Da) with a spectrum dialysis bag against reverse osmosis water for at least 3 days. The mPEG-poly(Ala) diblock copolymers were dried through lyophilization and stored for later use. The polymer has a molecular weight of 4600 Da with an approximate composition of mPEG2000-(Ala)_31_. Aqueous solution of this polymer at 5 wt% showed a phase transition from sol to gel at 34 °C as accessed via rheological measurements. The mPEG-poly(Ala) hydrogel has favorable biocompatibility with MIN6 cells, and degrades slowly [23].

### 2.3. Culture of MIN6 Cells 

MIN6 cells were cultured in DMEM medium supplemented with 1% penicillin-streptomycin and 15% heat-inactivated fetal bovine serum (FBS). The MIN6 cells were placed in 10 cm^2^ tissue culture dishes and incubated at 37 °C under 5% carbon dioxide. Media was changed every 3 days and cells were passaged weekly [23,28].

### 2.4. SubcutaneousTransplantation of MIN6 Cells

The animal experiment protocol was approved by the hospital Institutional Animal Care and Use Committee (No. 2018071901). Nude mice aged 8–12 weeks were purchased from the National Laboratory Animal Center, Taipei, Taiwan, and used as recipients of transplantation. 5.7 × 10^6^ MIN6 cells were first incubated overnight with CSPIO (10 μg/mL) in a 10-cm dish [28]. Cells were washed with DMEM medium before transplantation. 5 × 10^6^ cells in the 100 μL 5 wt% (mPEG)-poly(Ala) hydrogel solution and 20 µL RPMI medium were injected into bilateral flanks of each mouse (n = 5) [23,28]. Recipients’ body weights were measured and blood glucose values were checked by a glucose meter (Accu-Chek Guide, Roche, Mannheim, Germany) at least once per week in the first 2 weeks and twice per week thereafter post-transplantation.

### 2.5. Histological Analysis of Hydrogel-Embedded MIN6 Cell Grafts

To match the time points in our previous imaging studies [28], two grafts of hydrogel-embedded MIN6 cells in each mouse were removed at 8, 14, 21, 29 and 36 days after transplantation, respectively. They were fixed in a formalin solution and processed for paraffin embedding and sectioning. Graft sections were stained for iron with Prussian blue [28], for β-cells with an anti-insulin antibody [23,28], for cell proliferation with an anti-Ki67 antibody and for blood vessels with anti-CD31 [23] and anti-SMA antibodies [27].

## 3. Results and Discussion

### 3.1. Changes of Nude Mouse Recipients’ Blood Glucose and Body Weight after Subcutaneous Transplantation of Hydrogel-Embedded MIN6 Cells

**Figure 1:**
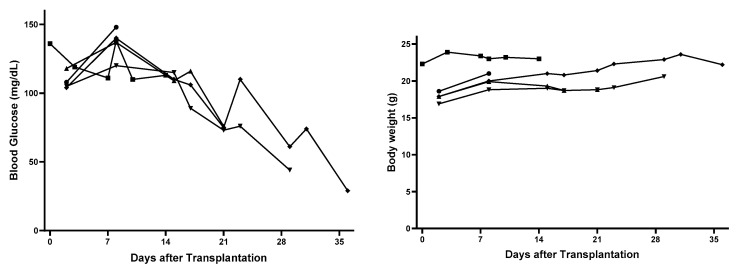
Changes of nude mouse recipients’ blood glucose (**left** panel) and body weight (**right** panel) after subcutaneous transplantation of 5 × 10^6^ (mPEG)-poly(Ala) hydrogel-embedded MIN6 cells. Each line indicates data from individual recipient mouse.

After subcutaneous transplantation of CSPIO-incubated and (mPEG)-poly(Ala) hydrogel-embedded MIN6 cells, nude mouse recipients’ blood glucose gradually decreased toward hypoglycemia, with the ranges of 120–148 mg/dL (n = 5) at day 8, 109–115 mg/dL (n = 3) at day 15, 73–76 mg/dL (n = 3) at day 21, 44–61 mg/dL (n = 2) at day 29, and 29 mg/dL (n = 1) at day 36 (left panel in Figure 1). In contrast, the body weight changes were insignificant, with the ranges of 18.8–21 g (n = 5) at day 8, 19–21 g (n = 3) at day 15, 18.8–21.4 g (n = 3) at day 21, 20.6–22.9 g (n = 2) at day 29 and 22.2 g (n = 1) at day 36 (right panel in Figure 1). Due to the limited supply of (mPEG)-poly(Ala) hydrogels, there was only 1 mouse used at each time point. Even though, we did find a substantial difference in their blood glucose values between those of “8 and 14 days” (n = 5) and “21, 29 and 36 days” (n = 3), namely 109–148 mg/dL in the former vs. 29–76 mg/dL in the latter. Clearly, there was a trend of decline in recipients’ blood glucose at day 15 and thereafter, indicating the hyperfunction of our implants.

The nude mouse is born without a thymus which is required for the generation of mature T lymphocytes. Therefore, they are unable to reject grafted MIN6 cells. Since MIN6 cells secrete insulin [24], it is expected that nude mouse recipients’ blood glucose fell and eventually to hypoglycemic levels. Our finding is consistent with that of Sobel et al. who found hypoglycemia (blood glucose <40 mg/dL) developed in all nude mice transplanted with 1 × 10^6^ MIN6 cells in Matrigel within 2 weeks [26]. In contrast, nude mice transplanted with MIN6 cells within HyStem-C developed hypoglycemia slower than those with MIN6 cells within Matrigel [26]. Moreover, high-passage min6 cells have impaired insulin secretion [30]. Therefore, the later development of hypoglycemia (between 21–29 days) in our recipients can be explained by the use of different hydrogels and/or passages of MIN6 cells. 

### 3.2. Vascularization of Subcutaneous Hydrogel-Embedded MIN6 Cell Grafts 

**Figure 2:**
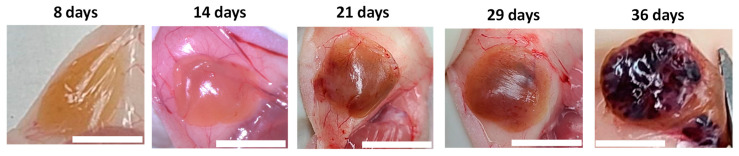
Subcutaneous grafts of (mPEG)-poly(Ala) hydrogel-embedded MIN6 cells. 5 × 10^6^ MIN6 cells were transplanted in subcutaneous tissues of nude mice and grafts were retrieved at 8, 14, 21, 29 and 36 days after transplantation. Scale bar: 1 cm.

**Figure 3:**
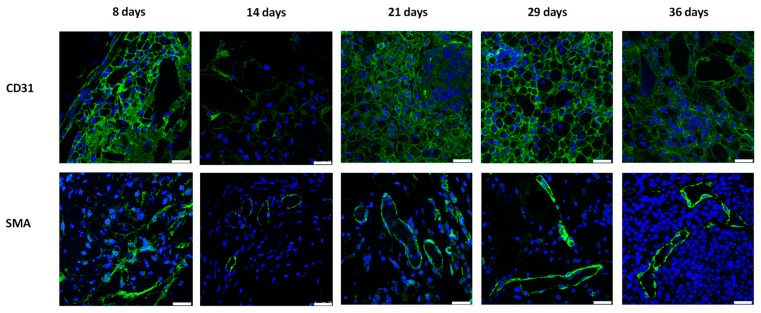
CD31 (upper panel, green color) and SMA (lower panel, green color) stainings of subcutaneous (mPEG)-poly(Ala) hydrogel-embedded MIN6 cell grafts. 5 × 10^6^ MIN6 cells were transplanted in subcutaneous tissues of nude mice and grafts were retrieved at 8, 14, 21, 29 and 36 days after transplantation. Scale bar: 25 μm.

**Figure 4:**
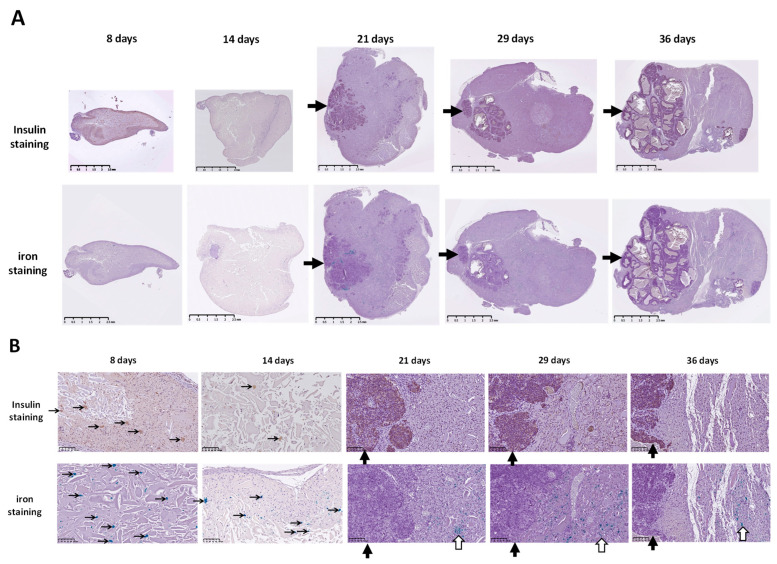
Insulin (brown color) and iron (blue color) staining of subcutaneous (mPEG)-poly(Ala) hydrogel-embedded MIN6 cell grafts. 5 × 10^6^ MIN6 cells were transplanted in subcutaneous tissues of nude mice and grafts were retrieved at 8, 14, 21, 29 and 36 days after transplantation. (**A**) 1.25×, scale bar: 2.5 mm; (**B**) 20×, scale bar: 100 μm. Thick black arrows indicate clusters of insulin-positive cells. Thick white arrows indicate clusters of insulin- and iron-positive cells. Thin black arrows indicate scattered insulin- or iron-positive cells.

**Figure 5:**
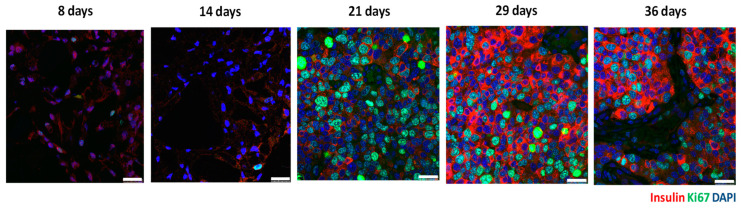
Insulin (red color) and ki67 (green color) immunofluorescence staining of subcutaneous (mPEG)-poly(Ala) hydrogel-embedded MIN6 cell grafts. 5 × 10^6^ MIN6 cells were transplanted in subcutaneous tissues of nude mice and grafts were retrieved at 8, 14, 21, 29 and 36 days after transplantation. Scale bar: 25 μm.

As shown in Figure 2, the color of subcutaneous MIN6 cell grafts changed from yellow at day 8 and 14, to brown at day 21 and 29, and finally black at day 36. As with other tumors, MIN6 cell line can induce their neovasculature by secreting angiogenic factors [31]. Thus, we speculated that these color changes are due to the progressive increase of local blood vessels. To confirm it, we stained grafts with anti-CD31 and anti-SMA antibodies. CD31 and SMA are present on endothelial cells and in vascular smooth muscle cells, respectively. Positive CD31 and SMA stainings indicate the presence of vascularization [23,27]. In Figure 3, we observed prominent CD31 and SMA stainings in 8-, 21-, 29- and 36-day MIN6 cell grafts. Less CD31 and SMA stainings at day 14 may be due to less tumor vessels induced by fewer MIN6 cells in the graft (Figure 4 and Figure 5). Previously, we showed positive loop-shaped CD31 staining in the subcutaneous graft of mPEG-poly(Ala) hydrogels without MIN6 cells at 7 and 14 days after implantation [23]. This indicates that hydrogels alone can induce new blood vessels at the subcutaneous site. In the present study, we further demonstrated that this neovascularization was present and persisted in hydrogel-embedded MIN6 cell grafts for up to 36 days. Here, CD31 also stained tumor blood vessels and showed positive staining around cells. The different distribution of our CD31 and SMA staining is because the tumor vasculature contained only a single layer of endothelium [32]. Since the subcutaneous site is poorly vascularized, the promotion of neovascularization would be beneficial for the graft survival.

### 3.3. Histological Evolution of Subcutaneous Hydrogel-Embedded MIN6 CELL GRAFTS

Previously, we developed the contrast agent CSPIO [29] and successfully applied it in long-term imaging transplanted MIN6 β-cells [28], porcine neonatal pancreatic cell clusters [33], as well as mouse islet iso- [34,35] and allo-grafts [35,36]. CSPIO contains iron particles that present hypointensity in MR images and, in histology, can be detected by Prussian blue staining [28,33,34,35,36]. Therefore, in the present study, we preincubated MIN6 cells with CSPIO before transplantation and then used iron staining to detect CSPIO-labeled cells in the graft. We observed that insulin-positive and iron-positive cells (i.e., originally transplanted MIN6 cells) were scattered in the graft (indicated by thin black arrows in Figure 4B) at 8 and 14 days. Then, clusters of insulin-positive cells without iron-positive cells (i.e., MIN6 cells grown after transplantation) appeared in the grafts at 21 days and persisted at 29 and 36 days (indicated by thick black arrows in Figure 4A,B). In our previous MIN6 graft imaging study, the bioluminescence images showed a stronger signal at 22 and 36 days compared to 13 days and the MR signals turned from hypointensity at day 21 to hyperintensity at day 29 [28] Above-mentioned MIN6 graft histology corresponds to these image results. Interestingly, clusters of insulin-positive cells without iron-positive cells located separately from clusters of insulin-positive cells and iron-positive cells in grafts (indicated by thick white arrows in Figure 4B), implying the former are neogrowth after transplantation. Sobel et al. also found large clusters of insulin-stained cells in nude mouse MIN6 grafts [26]; however, these cells could not be recognized as neogrowth or originally transplanted MIN6 cells. Accordingly, CSPIO and other nontoxic iron-containing nanoparticles can be used not only as contrast agents for MRI but also as tracers for cell transplantation. To confirm the tumor formation of transplanted MIN6 cells, we further stained the graft with cell proliferation marker ki67. Strong ki67 and insulin staining in the graft appeared at day 21 and 29, respectively (Figure 5), suggesting MIN6 cells proliferate to form neogrowth from day 21. Although there were only two graft samples obtained at each time point, we did find different patterns in the graft histology between those of “8 and 14 days” (n = 4) and “21, 29 and 36 days” (n = 6), including insulin-positive cells were scattered in the former but in cluster in the latter where prominent ki67 staining was observed. Overall, neogrowth from originally transplanted MIN6 cells results in those distinctive bioluminescence and MR images in our previous observations [28]. 

In the present study, we demonstrated that mPEG-poly(Ala) hydrogels were helpful for MIN6-cell growth at the subcutaneous site in nude mice. It will be interesting to explore alternative hydrogels or investigate the effects of different cell types on graft survival and neovascularization. To apply β-cell lines for diabetes treatment, several issues should be considered. Since MIN6 cells are derived from a mouse insulinoma, neogrowth after implantation was expected. However, uncontrolled cell proliferation and insulin oversecretion should be avoided after transplantation. Previously, Tsujimura et al. used a combination of the herpes simplex virus thymidine kinase gene and ganciclovir to regulate the proliferation and function of MIN6 cells after transplantation in diabetic mice [37]. The other concern is immune rejection. Since MIN6 cells are derived from a B6 mouse insulinoma, they will be rejected when transplanted into other strains or species. By using microencapsulated MIN6 cells, Kawakami et al. showed no evidence of inflammatory cells infiltrated within or around the microbeads at 1 week after subcutaneous xenotransplantation [38]. 

## 4. Conclusions

In previous research, we effectively employed noninvasive MR and bioluminescence imaging to identify and track mPEG-poly(Ala) hydrogel-encased MIN6 cells at a subcutaneous location for up to 64 days. In the present study, we noted insulin- and iron-positive cells dispersed throughout the graft at both 8 and 14 days. However, by day 21, groups of insulin-positive and iron-negative cells emerged within the grafts, persisting from that point forward, which suggests new growth of MIN6 cells. Moreover, we detected proliferating MIN6 cells displaying intense ki67 staining in grafts at 21, 29, and 36 days. Our findings reveal that the initial transplanted MIN6 cells begin to multiply at day 21, resulting in distinctive bioluminescence and MR images. In addition, to the best of our knowledge, we are the first to use CSPIO a tracer to differentiate the neogrowth from originally transplanted cells in histology.

## Data Availability

Not applicable.
